# Molecular subtyping of metastatic renal cell carcinoma: implications for targeted therapy

**DOI:** 10.1186/1476-4598-13-39

**Published:** 2014-02-26

**Authors:** Lisha Wang, Sean R Williamson, Mingsheng Wang, Darrell D Davidson, Shaobo Zhang, Lee Ann Baldridge, Xiang Du, Liang Cheng

**Affiliations:** 1 Department of Pathology, Fudan University Shanghai Cancer Center, Shanghai, China; 2 Department of Pathology and Laboratory Medicine, Indiana University School of Medicine, 350 West 11th Street, IU Health Pathology Laboratory Room 4010, Indianapolis, IN 46202, USA; 3 Department of Pathology and Laboratory Medicine, Henry Ford Health System, Detroit, MI, USA

**Keywords:** Kidney, Renal cell carcinoma, Metastasis, Targeted therapy, Molecular cytogenetics, Fluorescence in situ hybridization

## Abstract

**Background:**

Renal cell carcinoma (RCC) is known for its ability to metastasize synchronously or metachronously to various anatomic sites. Distinguishing histologic subtypes of metastatic RCC has become increasingly important, as prognosis and therapy can differ dramatically between subtypes. We propose a combination of immunohistochemistry (IHC) and molecular cytogenetics for subtyping metastatic RCC in light of these potential therapeutic implications.

**Results:**

Specimens from 103 cases of metastatic RCC were retrieved, including 32 cases originally diagnosed as metastatic clear cell renal cell carcinoma (CCRCC), 8 as metastatic papillary renal cell carcinoma (PRCC), and 63 metastatic RCC without a specific subtype. Immunohistochemistry was performed with antibodies against cytokeratin 7 (CK7) and alpha-methylacyl-CoA racemase (AMACR). Dual color interphase fluorescence in situ hybridization was utilized to assess for deletion of chromosome 3p and trisomy of chromosomes 7 and 17 in all tumors. Chromosome 3p deletion was detected in 41% of all metastatic RCC specimens, and trisomy of chromosomes 7 and/or 17 was detected in 16%. Of metastatic CCRCC, chromosome 3p deletion was detected in 63%. Of metastatic PRCC, 75% showed trisomy of chromosomes 7 and/or 17. Of the tumors not previously classified, 6% were positive for CK7, and 64% were positive for AMACR; 35% showed chromosome 3p deletion, and 16% showed trisomy of chromosomes 7 and/or 17. Combined analysis of immunohistochemistry and cytogenetics enabled reclassification of 52% of these metastatic tumors not previously classified.

**Conclusion:**

Our findings support the utility of immunohistochemistry and cytogenetics for subtyping metastatic RCC.

## Introduction

Renal cell carcinoma (RCC) comprises a heterogeneous group of epithelial neoplasms with diverse biologic behaviors and variable clinical outcomes. RCC is the most lethal of the urologic malignancies. Between 20% and 30% of patients with RCC have metastatic disease at the time of diagnosis, and another 30% subsequently develop metastasis after resection [1-3]. The majority of tumors are of the clear cell (CCRCC) subtype (70%-75%), characteristically harboring abnormalities of the von Hippel-Lindau (*VHL*) gene, located at chromosome 3p25 [3-9]. Defects in VHL expression result in constitutive activation of the hypoxia-inducible factor (HIF) pathway and overexpression of vascular endothelial growth factor (VEGF), platelet-derived growth factor (PDGF), and other products. Inactivation of the *VHL* gene also enhances tumor cell growth though the mammalian target of rapamycin (mTOR) pathway [8,10-12]. In contrast, papillary renal cell carcinoma (PRCC) is the most common non-clear cell subtype of RCC, accounting for 10%-15% of tumors. PRCC is associated with activation of the MET pathway in a subset of tumors, resulting in a cascade of intracellular signaling leading to tumor cell growth, angiogenesis, migration and invasion [6,13,14].

Knowledge of these gene pathways has enabled novel approaches to the management of metastatic RCC [15-17]. Currently, clinical trials with targeted therapeutic strategies for both metastatic CCRCC and PRCC have been intensively planned and carried out [6,13,18-26]. Although recent advances have improved patient outcomes [20,27-29], these targeted agents are not without toxic effects [30,31]. Optimizing the clinical outcome and knowing when to persist with these therapies highlight the need for accurate RCC subtyping.

Histopathologic examination of a completely resected primary tumor is often sufficient for tumor subtyping, as a component with prototypical morphologic features can usually be readily appreciated. However, in the metastatic setting, it is often challenging to discriminate between subtypes of RCC based on morphology alone, particularly since metastatic foci are often sampled only by core needle biopsy and are often preferentially composed of high-grade tumor. Immunohistochemical analysis is valuable to identify the histogenetic origin of metastatic malignancy [32]. Nevertheless, its use for discriminating different histologic subtypes is limited and rarely applied in prospective treatment outcome studies. A cytogenetic hallmark of CCRCC is loss of chromosome 3p, which distinguishes it from other RCC subtypes [7,8,33]. PRCC frequently exhibits chromosomal polysomies, of which trisomy of chromosomes 7 and/or 17 are the most consistent and characteristic [7,8,34]. Because CCRCC and PRCC show different immunophenotypes and different characteristic cytogenetic abnormalities, we sought to combine these two ancillary tests in an effort to reduce ambiguity in subtyping of metastatic RCC. Immunophenotypes of 103 cases of metastatic RCC were analyzed in conjunction with cytogenetic characteristics as determined by fluorescence in situ hybridization (FISH), in order to improve classification of these neoplasms.

## Patients and methods

### Patients

One hundred three cases of metastatic RCC diagnosed between 2007 and 2013 were retrieved from the archives of the Department of Pathology of the Indiana University School of Medicine. The histologic type was established, when possible, according to the 2004 WHO classification [3]. The hematoxylin and eosin slides of these cases were reviewed, and appropriate tumor blocks from metastatic sites were selected for immunohistochemical and cytogenetic studies. This research was approved by the Indiana University Institutional Review Board.

### Immunohistochemical staining

Immunohistochemistry was performed with the following antibodies: cytokeratin 7 (CK7; monoclonal mouse anti-human CK7 antibody, OV-TL 12/30, prediluted; Dako Corp.) and alpha-methylacyl-CoA-racemase (AMACR/P504S, polyclonal rabbit anti-human antibody, 13H4 clone, prediluted; Dako Corp.). Diaminobenzidine (3, 3-diaminobenzidine) was used as the chromogen. Immunostaining was performed on the DAKO Autostainer Plus. Positive and negative controls were stained concurrently and showed appropriate immunostaining. The extent of immunohistochemical staining was evaluated microscopically. Labeling for CK7 and AMACR was considered positive when moderate to strong staining was present in greater than 20% of tumor cells.

### Fluorescence in situ hybridization

Fluorescence in situ hybridization (FISH) analysis was performed as described previously [5,7,34-37]. Briefly, multiple 4 μm sections were obtained from formalin-fixed paraffin-embedded tissue blocks containing neoplastic tissue. A hematoxylin and eosin-stained slide from each block was examined to identify areas of neoplastic tissue for FISH analysis. The slides were deparaffinized with 2 washes of xylene, 15 minutes each, and subsequently washed twice with absolute ethanol, 10 minutes each, and then air-dried in a fume hood. Next, the slides were treated with 0.1 mM citric acid (pH 6.0) (Zymed, South San Francisco, CA) at 95°C for 10 minutes, rinsed in distilled water for 3 minutes, followed by a wash of 2x standard saline citrate for 5 minutes. Digestion was performed by applying 0.4 mL of pepsin (5 mg/mL in 0.1 N HCl/0.9 NaCl) (Sigma, St Louis, MO) at 37°C for 40 minutes. The slides were rinsed with distilled water for 3 minutes, washed with 2x standard saline citrate for 5 minutes and air-dried. The chromosomal probe directed against 3p25 (RP11-572 M14) was obtained from Empire Genomics (Empire Genomics, Buffalo, New York). Chromosome enumeration probes (CEP) for chromosomes 3, 7, and 17 were obtained from Vysis (Abbott, Downers Grove, IL).

Deletion of chromosome 3p was assessed using a probe cocktail containing BAC clone probe to chromosome 3p25 (RP11-572 M14, Green) and CEP3 (Orange). Chromosome 7 and 17 alterations were assessed using a probe cocktail containing probe CEP7 (Green) and CEP17 (Orange). The 3p25/CEP3 probe set and the CEP7/CEP17 probe set were diluted with tDenHyb2 (Insitus, Albuquerque, NM) in ratios of 1:50 and 1:100, respectively.

Analysis was performed in a manner similar to that previously described [5,7,34-37]. In brief, for each slide 100 to 150 nuclei from tumor tissue were scored for probe signals under the fluorescence microscope with x1000 magnification. The method of analysis for 3p25 deletion was based on previous studies of chromosome deletions at 1p and 19q in oligodendrogliomas [38,39]. The cutoff value for 3p deletion was defined as a 3p25/CEP3 ratio of ≤0.7. Definitions of chromosomal trisomy for chromosomes 7 and 17 were based on the Gaussian model and were related to the nonneoplastic renal cortex control cell signals. The cutoff values were set for each probe at the mean plus 3 standard deviations of the control values. Chromosome 3p deletion was considered to be characteristic of CCRCC, whereas trisomy of chromosomes 7 and/or 17 was considered characteristic of PRCC [7].

## Results

### Clinicopathologic characteristics

A total of 103 metastatic RCC specimens were included in the study. Of these, 32 tumors were originally classified as metastatic CCRCC, and 8 were originally classified as metastatic PRCC, based on a constellation of typical morphologic features and known histologic classification of the primary tumor. In the remaining 63 cases of metastatic RCC, the tumor histologic subtype was unknown or uncertain, based on lack of availability of tissue material from the primary tumor for comparison (including primary tumors that were not resected or those diagnosed an another institution) and equivocal morphologic features in the metastatic lesion. Seventy-five patients were male and 28 were female. The patients’ ages ranged from 28 to 87 years (median 63 years). The metastatic sites included: bone (n = 27), lung (n = 19), abdominal sites (n = 17), liver (n = 7), pleura (n = 7), soft tissue (n = 7), pancreas (n = 3), gastrointestinal tract (n = 3), mediastinum (n = 3), gallbladder (n = 2), thyroid (n = 2), and other organs or tissues (n = 6).

### Immunohistochemistry

**Figure 1:**
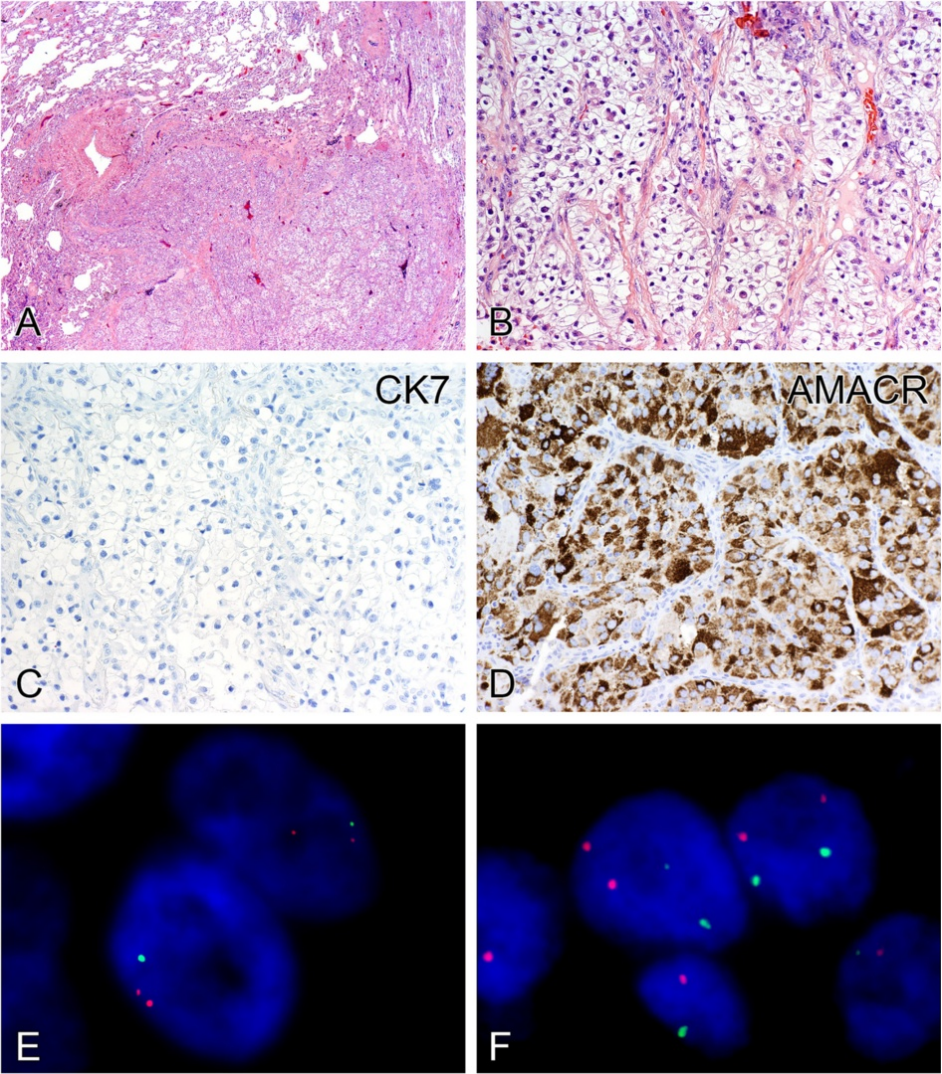
**Metastatic clear cell renal cell carcinoma.** A metastatic tumor nodule in the lung **(A)** was composed of solid, nested, and tubular arrangements of cells with clear cytoplasm **(B).** The tumor cells showed negative immunoreactivity for CK7 **(C)** but strong reactivity for AMACR **(D)**. Dual-color fluorescence in situ hybridization demonstrated chromosome 3p deletion, as indicated by the presence of a single 3p25 signal (green) with two chromosome 3 centromere signals (red) per cell **(E)**. Tumor nuclei showed two green signals for chromosome 7 and two red signals for chromosome 17 **(F)**, disomic patterns.

**Figure 2:**
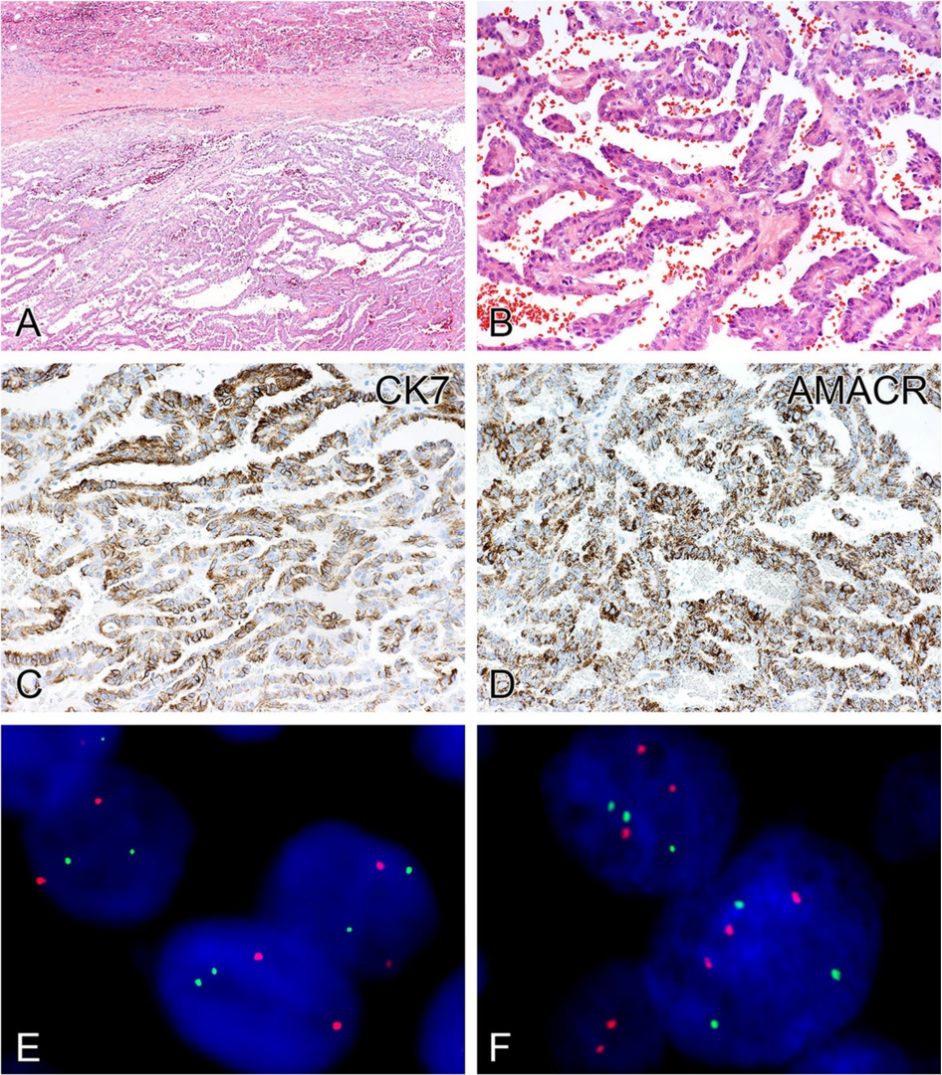
**Metastatic papillary renal cell carcinoma.** A metastatic tumor nodule in the liver exhibited characteristic papillary architecture **(A)**, composed of fibrovascular cores lined by cells with eosinophilic cytoplasm **(B).** The tumor cells labeled strongly with antibodies to CK7 **(C)** and AMACR **(D)**. Dual-color fluorescence in situ hybridization revealed intact chromosome 3p, as indicated by two green signals (3p25) and two chromosome 3 centromeric red signals (CEP3) **(E)**. Dual-color fluorescence in situ hybridization demonstrated trisomy of chromosomes 7 and 17, as evident by three green signals (CEP7) and three red signals (CEP17) in each tumor cell nucleus **(F)**, supporting the classification of papillary renal cell carcinoma.

Overall, 7% (7/103) of all metastatic RCC showed positive immunohistochemical staining for CK7, and 57% (59/103) showed immunoreactivity for AMACR (Table 1, Figures 1 and 2). Of the tumors originally classified as metastatic CCRCC, none were positive for CK7 and 34% (11/32) were positive for AMACR. Of the tumors originally classified as metastatic PRCC, 38% (3/8) were positive for CK7, and 100% (8/8) were positive for AMACR. Of the RCCs with an uncertain or unknown histologic subtype, 6% (4/63) were positive for CK7, and 64% (40/63) were positive for AMACR (Table 1). Of the CK7-positive tumors, 86% (6/7) also demonstrated trisomy of chromosomes 7 and/or 17 by FISH.

**Table 1:** Summary of IHC markers and cytogenetic abnormalities in different metastatic RCC subtypes

**mRCC Subtypes (n)**	**IHC**		**Cytogenetics**	
	**CK7**	**AMACR**	**3p deletion**	**Trisomy 7 and/or 17**
CCRCC (32)	0% (0/32)	34% (11/32)	63%(20/32)	0% (0/32)
PRCC (8)	38% (3/8)	100% (8/8)	0% (0/8)	75% (6/8)
Not classified (63)	6% (4/63)	63% (40/63)	35% (22/63)	16% (10/63)
Total (103)	7% (7/103)	57% (59/103)	41% (42/103)	16% (16/103)

*Abbreviations*: mRCC, metastatic renal cell carcinoma; CCRCC, clear cell renal cell carcinoma; PRCC, papillary renal cell carcinoma; IHC, immunohistochemistry; AMACR, alpha-methylacyl-CoA racemase.

### Cytogenetics

**Figure 3:**
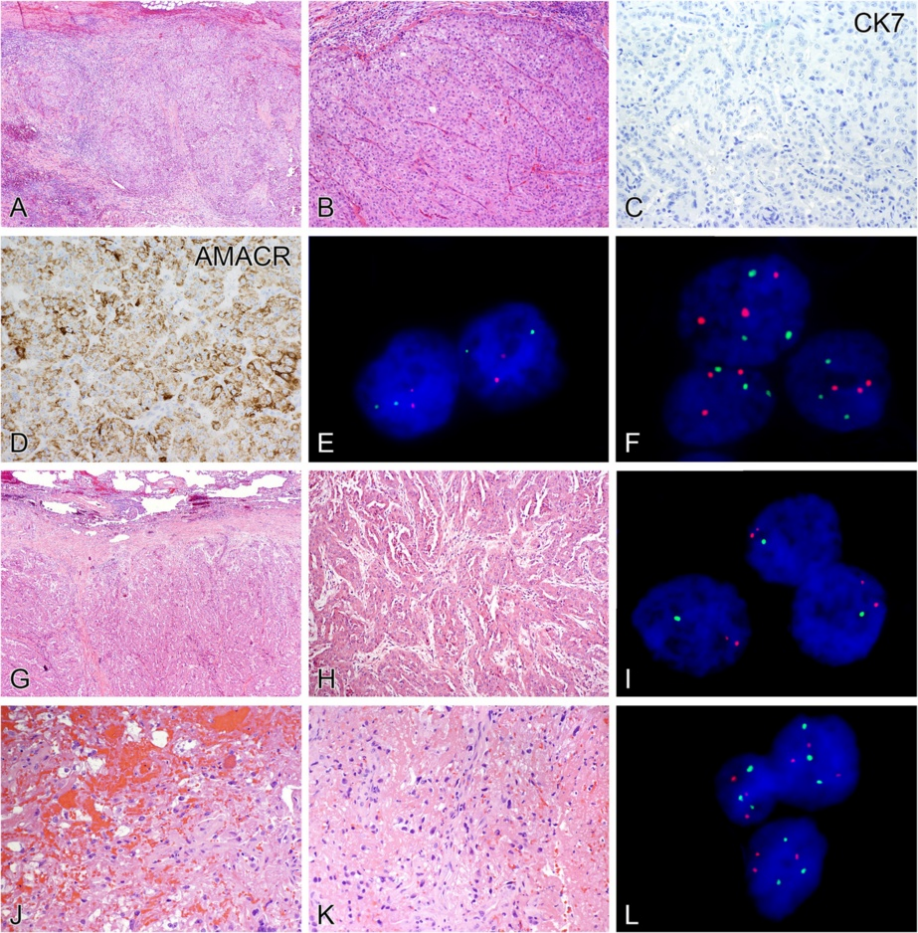
**Metastatic renal cell carcinomas not previously classified. A metastatic tumor nodule involving a lymph node (A) exhibited a nested to sheet-like architecture (B).** Although morphologic features supported a diagnosis of metastatic renal cell carcinoma, the precise tumor subtype was not initially apparent. The tumor cells did not label for CK7 **(C)**, but demonstrated moderately strong granular cytoplasmic reactivity for AMACR **(D)**. Dual-color fluorescence in situ hybridization revealed intact chromosome 3p **(E)** as indicated by two green signals (3p25) and two red chromosome 3 centromeric signals (CEP3). FISH demonstrated trisomy of chromosomes 7 and 17, as indicated by three green signals (CEP7) and three red signals (CEP17) per nucleus **(F)**, supporting reclassification of this tumor as papillary renal cell carcinoma. Another metastatic renal cell carcinoma involving the lung showed architectural and cytologic features not initially recognizable as those of a particular renal cell carcinoma subtype **(G)**. The tumor exhibited a mixture of solid, trabecular, and tubulopapillary growth, lined by cells with eosinophilic cytoplasm **(H)**. Dual-color fluorescence in situ hybridization demonstrated a characteristic 3p25 deletion as indicated by one green signal (3p25) and two chromosome 3 centromere red signals (CEP3) per cell **(I)**, supporting subtyping as high-grade manifestation of clear cell renal cell carcinoma. A third metastatic lesion involving bone **(J)** contained cells with clear cytoplasm and prominent nucleoli, dispersed in a hemorrhagic and fibrinous **(K)** background. These morphologic findings raised the possibility of metastatic clear cell renal cell carcinoma. However, FISH revealed trisomy of chromosomes 7 and 17 **(L)**, as demonstrated by three green signals (CEP7) and three red signals (CEP17) per nucleus. These findings were in line with the patient’s history of papillary renal cell carcinoma resected via radical nephrectomy many years prior, despite the morphologic appearance raising the possibility of clear cell renal cell carcinoma.

Chromosome 3p deletion was detected in 41% (42/103) of all metastatic RCC cases, and trisomy of chromosomes 7 and/or 17 was detected in 16% (16/103), of which 14 exhibited trisomy of chromosome 7 and 12 exhibited trisomy of chromosome 17 (Table 1). Of the tumors originally classified as metastatic CCRCC, chromosome 3p deletion was detected in 63% (20/32). Of the tumors originally classified as metastatic PRCC, 75% (6/8) showed trisomy of chromosome 7 and/or 17. Deletions of chromosome 3p and trisomy of chromosomes 7 and/or 17 were mutually exclusive findings in these metastatic CCRCC and PRCC cases. Of the RCCs not previously classified, 35% (22/63) were found to have chromosome 3p deletion, and 16% (10/63) were found to have trisomy of chromosome 7 and/or 17 (Table 1). Therefore 51% (32/63) of the metastatic RCCs that were previously not classified could be subtyped based on cytogenetic alterations: The tumors with chromosome 3p deletion (35%, 22/63) were reclassified as metastatic CCRCC, and the tumors with trisomy 7 and/or 17 (16%, 10/63) were reclassified as metastatic PRCC (Table 1, Figure 3).

Of the 22 tumors reclassified based on FISH results as metastatic CCRCC, none were positive for CK7, and 46% (10/22) were positive for AMACR. Of the 10 cases reclassified as metastatic PRCC, 30% (3/10) were positive for CK7, and 100% (10/10) were positive for AMACR. One tumor was positive for CK7 but lacked any cytogenetic abnormality detected by these methods. If this tumor is regarded as PRCC based on positivity for CK7, then 52% (33/63) of the metastatic RCCs that were not previously subtyped could be reclassified based on a combination of FISH and immunohistochemistry.

## Discussion

RCC is known for its ability to metastasize either synchronously or metachronously to a variety of anatomic sites. In current practice, distinguishing histologic subtypes of metastatic RCC has become increasingly important, as different subtypes portend divergent prognoses and are managed with disparate treatment algorithms. Histologic features enable accurate classification of most primary tumors. However, overlapping morphologic findings between some categories of renal neoplasms can make subclassification difficult, particularly in the metastatic setting, in which biopsy material may be limited and high-grade morphology may obscure prototypical histopathologic architectural and cytologic features. Additionally, recent molecular insights into the clonal evolution of metastatic RCC have revealed substantial heterogeneity in genetic alterations in different regions of metastatic deposits and within different regions of the primary tumor [40]. In the era of targeted therapies, different histologic subtypes of metastatic RCC have relevance in selecting patients for enrollment in clinical trials and in evaluation for salvage therapy [1,8,41]. Currently, pivotal studies using targeted drugs have largely focused on patients with clear cell RCC. Patients with tumors of other non-clear cell histologic subtypes have been less extensively studied [27]. In the present study, we evaluated the immunophenotypes of 103 metastatic RCCs, and correlated with the tumors’ cytogenetic characteristics using FISH. Combined analysis of immunohistochemistry and cytogenetics enabled reclassification of 52% (33/63) of metastatic RCCs for which the histologic subtype was originally unknown or uncertain. Our study establishes the utility of immunohistochemistry and cytogenetics for subtyping metastatic RCC, which may be of particular help toward selecting appropriate targeted therapies.

RCC is not a single disease; it is composed of a number of subtypes, each with unique histologic features, genetic alterations, clinical behavior, and response to therapy [1,7,8,42]. Nonetheless, histologic subtyping of RCC can be particularly problematic in the metastatic setting for a number of reasons: For one, tissue diagnosis of metastatic RCC is sometimes established prior to or in the absence of sampling the primary tumor. Conversely, identification of metastatic RCC sometimes follows resection of the primary tumor by a long intervening period. Further, metastatic RCC may preferentially exhibit high-grade morphology, lacking the characteristic cytologic and architectural features that are often admixed with higher-grade components in the primary tumor. Therefore, some metastatic RCCs present as a tumor of unknown origin, with a prior history of RCC unknown to treating oncologists or pathologists. In the metastatic context, core needle biopsies and fine needle aspiration specimens from metastatic RCC may be particularly challenging due to limited material for evaluation [43].

Advances in biologic and genetic understanding of RCC have led to specifically targeted treatments for metastatic RCC. For example, inhibition of targets in the HIF pathway has resulted in significant clinical responses in CCRCC [44,45]. Loss of activity of the Krebs cycle enzyme fumarase hydratase (FH) in some cases of papillary type II RCC may also result in HIF upregulation [46], potentially providing an avenue for utilization of similar treatments in patients with PRCC. Activation of the c-MET oncogene is characteristic of papillary RCC type I, particularly in the hereditary PRCC syndrome and a subset of sporadic PRCC [8]. This finding offers a clear opportunity to test newly developed inhibitors of this tyrosine kinase in this subset of RCC [44,47]. Although these effective biologic agents may be used in a more individualized approach to metastatic RCC therapy, their novelty infers a paucity of clinical data about their toxic effects or management of their therapy-limiting complications in the setting of metastatic RCC [30]. Therefore, histological subtyping of metastatic RCC significantly impact clinical decision making and therapeutic outcomes in these patients.

CK7 and AMACR have been proposed as markers to help distinguish PRCC from other RCC types, especially CCRCC [34,36,48-51]. Immunostaining for CK7 in CCRCC is usually negative or only focally positive, contrasting with more diffuse labeling for this protein in many PRCCs [50-53], particularly type I PRCC. Diffuse, strong AMACR expression is typical of PRCC (70-100%); however, reactivity has also been observed to a variable extent in 4-68% of CCRCC [49,50,54-58], sometimes less diffusely or associated with higher-grade tumor components. When evaluating these two markers for RCC, focus has been predominantly directed at primary tumors. We performed immunohistochemical staining for CK7 and AMACR in this series of 103 nonprimary cases to confirm their expression in metastatic RCC. None of the metastatic CCRCC met the study threshold for positive CK7 staining. Only 38% (3/8) of the metastatic PRCC and 6% (4/63) of the RCCs that were previously not classified were positive for CK7, suggesting that expression of this marker may be attenuated in metastatic RCC. CK7 was relatively specific for metastatic PRCC, although not as sensitive as AMACR, perhaps due to its expression in predominantly type I rather than type II tumors [59]. AMACR was detected in 23 of 28 metastatic CCRCC (82%) in one study [60], and 6 of 6 metastatic PRCC (100%) in another study [61]. In our study, AMACR was expressed by 57% (59/103) of all metastatic RCC, including 11 cases of metastatic CCRCC (34%, 11/32), all metastatic PRCCs (100%, 8/8), and 40 cases of RCC not previously classified (63%, 40/63). AMACR was relatively sensitive for metastatic PRCC, but its specificity was not high. Therefore, the value of CK7 and AMACR immunostaining alone is limited for accurately subtyping metastatic RCC.

Other immunohistochemical antibodies with emerging utility in the subclassification of RCC include those directed against carbonic anhydrase IX [62-64]. Since this enzyme is a downstream target of the VHL-HIF pathway [64-66], it is reported to exhibit diffuse, strong membranous reactivity by immunohistochemistry in CCRCC, in contrast to other subtypes of renal tumors, which typically exhibit focal or multifocal reactivity [63], sometimes juxtaposed to areas of ischemia or necrosis. However, other investigators have found intratumoral heterogeneity using this marker, particularly in high-grade or sarcomatoid tumors, such as those that might be encountered at a metastatic site [67]. Since tissue sampling of a metastatic tumor is also likely to be limited, the significance of positive reactivity for carbonic anhydrase IX may be uncertain compared to large samples from a completely resected tumor, in which extent of reactivity can be more readily assessed.

Interphase cytogenetic analysis has emerged as a powerful tool for diagnosis and classification of RCC [34,68-72]. The cytogenetic hallmark of CCRCC is loss of chromosome 3p, the chromosomal site of the *VHL* gene and other important loci involved in CCRCC tumorigenesis [8,33]. FISH analysis shows the characteristic chromosome 3p deletion in 60-90% of CCRCC cases [33,73]. In contrast, PRCC frequently exhibits chromosomal polysomies, of which trisomy of chromosomes 7 and/or 17 are the most consistent and characteristic [34]. The current study provides cytogenetic data for metastatic RCC involving a variety of anatomic sites. Chromosome 3p deletion was detected in 41% (42/103) of all metastatic RCC cases, and in 63% (20/32) of tumors originally diagnosed as metastatic CCRCC. Of tumors originally diagnosed as metastatic PRCC, 75% (6/8) showed trisomy of chromosomes 7 or 17. Of the metastatic RCCs that were not originally classified, 35% (22/63) additionally exhibited chromosome 3p deletion, facilitating reclassification as metastatic CCRCC. An additional 16% of these tumors (10/63) were found to have trisomy of chromosomes 7 and/or 17, supporting reclassification as metastatic PRCC. In this study, we found chromosome 3p deletion and trisomy 7 or 17 to be mutually exclusive in metastatic RCCs; however, other investigators have occasionally found chromosome 3p deletion to coexist with trisomy 7 or 17 in PRCC, such as in some type II PRCC, and some CCRCC [8,69,72,74]. Therefore, when both of these alterations are present in the same tumor, the findings should be interpreted with caution in supporting the diagnosis of a particular RCC subtype. One additional tumor was found to have no cytogenetic abnormality by FISH but positive expression of CK7 by immunohistochemistry. If this tumor is also considered to be PRCC based on this immunoreactivity pattern, 52% (33/63) of the metastatic RCCs that were previously not classified could be subtyped based on the combination of immunohistochemistry and FISH. A limitation of this study is that we assessed primarily only the two most common RCC subtypes, CCRCC and PRCC. However, a number of other RCC subtypes are now increasingly recognized [75], such as those associated with translocations involving MITF family genes [1,7,8,76-79]. Such neoplasms often exhibit overlapping morphologic features of CCRCC and PRCC, yet they are characterized by unique clinicopathologic, immunohistochemical and genetic alterations. In contrast to the 3p deletions and trisomy of chromosomes 7 and 17 in CCRCC and PRCC, respectively, FISH analysis has assumed a key role in confirming rearrangements involving the *TFE3* gene in such tumors [1,78,79] and to a lesser extent, the *TFEB* gene [80].

In summary, subtyping of metastatic RCC has become increasingly important with the emergence of novel therapies for specific tumor subtypes. Our data support the utility of a combined approach of immunohistochemistry and cytogenetics for subtyping metastatic RCC. Our findings may have important diagnostic and clinical implications in the era of personalized medicine, with the advent of target-specific therapeutics.

## Competing interests

The authors declare that they have no competing interests.

## Authors’ contributions

LW is responsible for the execution, data interpretation, data analyses and drafting of the manuscript. LAB performed immunostaining for the study. SRW, MW, DDD, SZ, XD, and LC contributed to conception and design of study, data preparation and analysis, manuscript drafting and revisions. All authors read and approved the final manuscript.
